# NF-κB regulates neuronal ankyrin-G via a negative feedback loop

**DOI:** 10.1038/srep42006

**Published:** 2017-02-09

**Authors:** Hans-Georg König, Robert Schwamborn, Silke Andresen, Sinéad Kinsella, Orla Watters, Beau Fenner, Jochen H. M. Prehn

**Affiliations:** 1 Department of Physiology and Medical Physics, Royal College of Surgeons in Ireland, 123 Saint Stephen’s Green, Dublin 2, Ireland; 2 Centre for the Study of Neurological Disorders, Royal College of Surgeons in Ireland, 123 Saint Stephen’s Green, Dublin 2, Ireland

## Abstract

The axon initial segment (AIS) is a neuronal compartment defined by ankyrin-G expression. We here demonstrate that the IKK-complex co-localizes and interacts with the cytoskeletal anchor protein ankyrin-G in immunoprecipitation and proximity-ligation experiments in cortical neurons. Overexpression of the 270 kDa variant of ankyrin-G suppressed, while gene-silencing of ankyrin-G expression increased nuclear factor-κB (NF-κB) activity in primary neurons, suggesting that ankyrin-G sequesters the transcription factor in the AIS. We also found that p65 bound to the *ank3* (ankyrin-G) promoter sequence in chromatin immunoprecipitation analyses thereby increasing *ank3* expression and ankyrin-G levels at the AIS. Gene-silencing of p65 or ankyrin-G overexpression suppressed *ank3* reporter activity. Collectively these data demonstrate that p65/NF-κB controls ankyrin-G levels via a negative feedback loop, thereby linking NF-κB signaling with neuronal polarity and axonal plasticity.

Expression of the cytoskeletal protein ankyrin-G is critical for the formation of the initial segment of mammalian central and peripheral axons (AIS), where it serves as the central scaffold^1^,^2^. More than 25 splice variants of ankyrin-G have been characterized in rodents with 270 and 480 kDa variants representing the most abundant isoforms at the AIS^1^,^3^,^4^.

Ankyrin-G protein isoforms are comprised of several membrane-binding and protein-protein interaction domains^1^,^3^. These allow ankyrin-G to act as a potent multi-domain scaffold, for instance to sequester sodium-channels and potassium-channels at the axon initial segment^5^,^6^,^7^. Accumulation of these ion channels and their accessory molecules is critical for spike initiation, form and frequency and hence represents a critical determinant of neuronal excitation^6^,^8^. Modulation of ankyrin-G levels and activity-dependent relocation is an important process as it regulates axonal excitability in a calcium-dependent manner^9^.

Ankyrin-G also co-operates with the spectrin-actin-network through interacting with βIV-Spectrin^2^,^10^ and forms cytoskeletal and cytosolic diffusion barriers at the initial segment^11^,^12^,^13^,^14^. These diffusion barriers are critical for axonal identity, as loss of ion channel accumulation^15^ and conversion of axons to dendrites occurs in response to ankyrin-G deletion^16^. This is also of pathophysiological significance as ischemic or traumatic injury to the axon may trigger calpain-dependent degradation of ankyrin-G^17^,^18^,^19^.

However, while ankyrin-G is crucial for neuronal excitability and axonal identity, little is known how ankyrin-G expression levels are regulated. Our understanding of factors regulating ankyrin-G transcription is currently limited to FoxJ1 which controls ankyrin-G aided progenitor to ependymal cell maturation^20^.

The transcription factor nuclear factor-κB (NF-κB) regulates the transcription of a large variety of genes in a cell-type and stimulus-dependent manner (reviewed in refs 21, 22, 23, 24, 25). We previously showed increased stability and decreased activity of NF-κB essential modulator (NEMO), the regulatory subunit of the NF-κB activating IκBα-kinase (IKK), following its interaction with the AIS-localized fibroblast growth factor homologous factor 1b (FHF1b^26^). We hypothesized that NF-κB is involved in homeostatic regulation of ankyrin-G expression levels. We here sought to establish a physiological role for the NF-κB pathway in regulating the axon initial segment, and demonstrate that NF-κB controls ankyrin-G levels at the AIS via a negative feedback-loop.

## Results

### The IKK-complex co-localizes and interacts with ankyrin-G *in vitro* and *in vivo*

**Figure 1:**
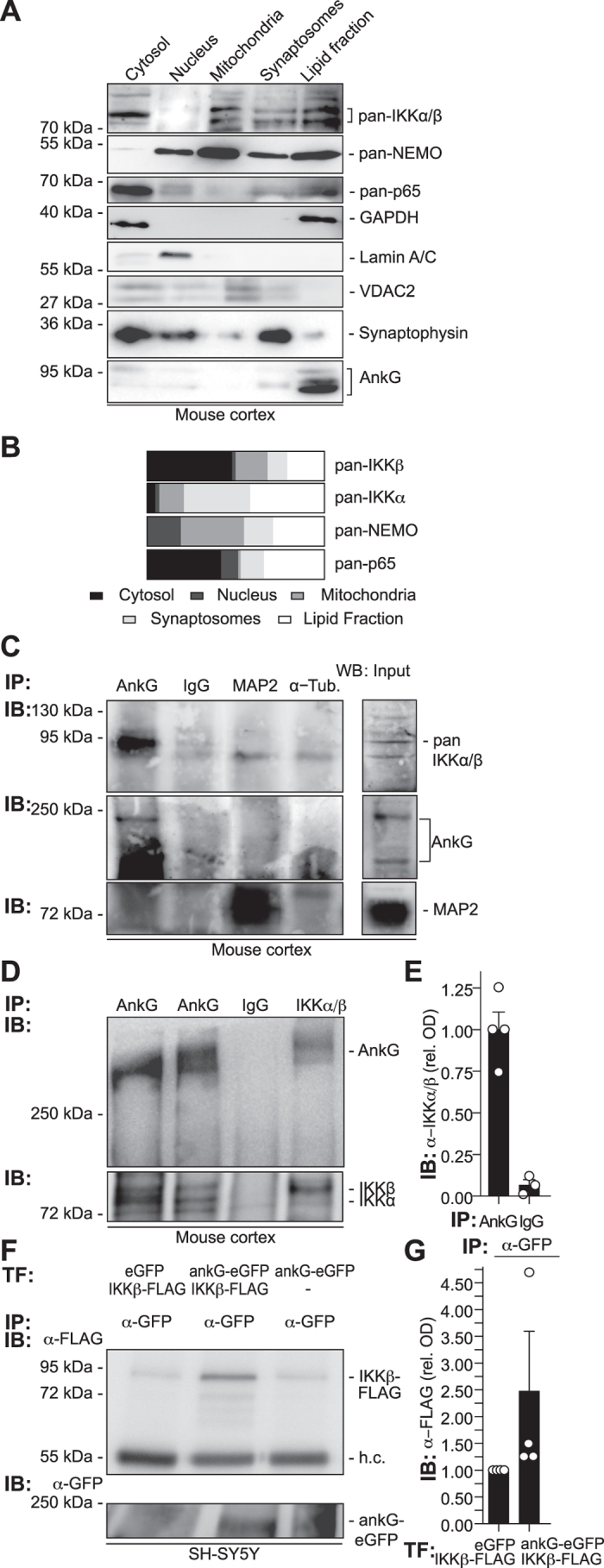
Ankyrin-G and the IKK-complex accumulate in synaptosomal and lipid fractions in the adult neocortex and co-immunoprecipitate. (**A**) Fresh adult mouse neocortex tissue was isolated and separated using gradient centrifugation. Proteins were extracted from cellular fractions and analyzed by Western blotting. Note the co-localization of pan-IKKα/β, NEMO and p65/NF-κB immunoreactivities to the ankyrin-G (clone 463, SCBT) positive lipid fraction. (**B**) Optical densities (OD) of protein immunoreactivity from all fractions were determined and mapped as percentage of total OD of all fractions in bar graphs. (**C**) Ankyrin-G and the IKK-complex co-immunoprecipitate from adult mouse cortical lysates. Mouse cortices were homogenized and minced in Na-Sucrose buffer. Equivalent amounts were used for immunoprecipitation (IP) using anti-ankyrin-G, control anti-IgG, anti-MAP2 or anti-α-Tubulin antisera, followed by immuno-blotting (IB) using anti-ankyrin-G (463), anti-pan-IKKα/β (H-470) and anti-MAP2 antibodies. Full-blot views including spliced supernatant control bands in Suppl. Fig. 1A. (**D**) For the reverse experiment, cortical tissue was processed and immunoprecipitated using a rabbit polyclonal antibody to IKKα/β (H-470, SCBT), a rabbit polyclonal anti-IgG antiserum served as control. Rabbit-polyclonal antibodies against ankyrin-G (S.E. Lux) and an alternative commercial antibody raised against ankyrin-G (H-215) were applied to additional samples. Following electrophoresis and immunoblotting, anti-ankyrin-G (S.E. Lux) and anti-pan-IKKα/β (H-470) antibodies were applied in Western-blotting. Full-blot views included in Suppl. Fig. 1B. (**E**) The relative OD of anti-IKKα/β immunoreactivity of all three Ankyrin-G IP’s (Fig. 1C,D and Suppl. Fig. 1C) was plotted against respective IgG-control IPs. (**F**) Anti-GFP antibodies pulled Flag-tagged IKKβ down from ankG-eGFP co-transfected, but not eGFP co-transfected cells. AnkyrinG-eGFP pull-down experiments were conducted following a 2-day transfection of SH-SY5Y cells with ankG-eGFP and IKKβ-FLAG vectors. Tag-specific antibodies were used to detect the proteins following Western-blotting procedure. (**G**) Quantification of anti-FLAG immunoreactivity (ODs representing IKK-β) depicted in (F) and three additional, similarly conducted pull-down experiments was determined.

**Figure 2:**
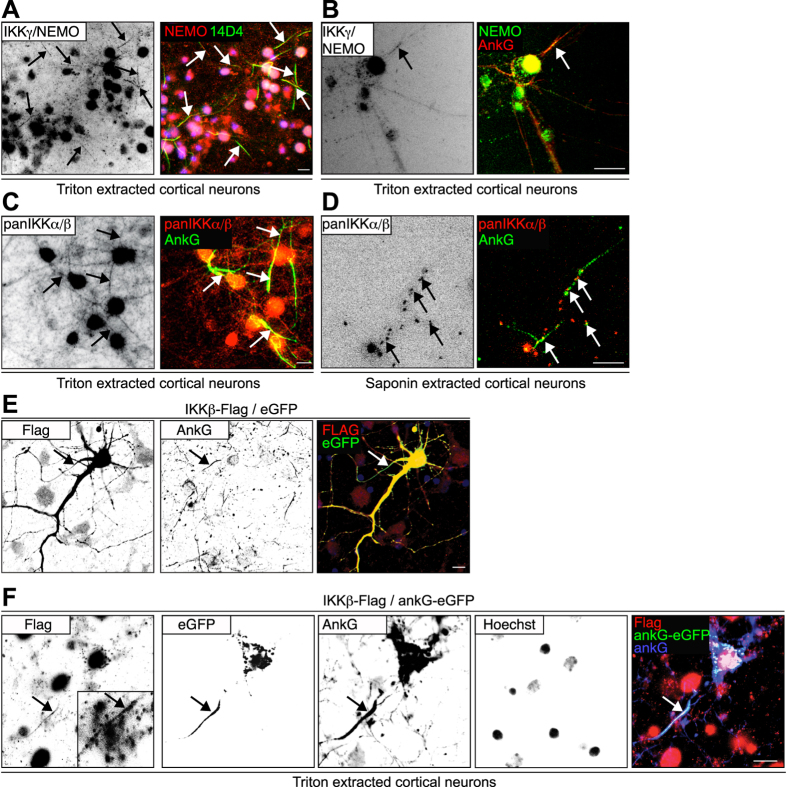
The IKK-complex co-localizes with ankyrin-G to the axon initial segment. (**A**–**D**) Cortical neurons were cultured until maturity *in vitro* and were cytosol-extracted for the visualization of cytoskeletal-associated proteins. They were incubated for 5 minutes in 1% (m/V) Triton-X-100 (A–C) or 0.5% saponin (D) in 10 mM Na_3_PO_4_-buffer (pH 7.4), 1 mM MgCl_2_, 3 mM CaCl_2_, 150 mM NaCl. Extractions were performed at 4 °C, prior to fixation and immunostaining. Anti-IKKγ/NEMO immunoreactivity is highlighted in red (A) or green (B), the AIS-specific marker^44^ antibody 14D4 (green,A) or anti-AnkG (red, B) were used to identify the AIS (green). (C,D) Extractions were similarly performed followed by immunolabeling using pan-IKKα/β - specific antibodies (red, rabbit polyclonal, H-470, SCBT) with the AIS labeled by anti-ankyrinG in green. Color overlays are depicted following background substraction and sharpening, arrows highlight AISs, scale bars, 10 μm. (**E**) IKKβ-Flag and eGFP vectors were transfected into mouse cortical neurons on DIV13 using Ca-precipitation. 2 days following transfection, neurons were fixed and immunolabeled using a rabbit polyclonal anti-Flag-specific antibody (Sigma, red), as well as a mouse monoclonal ankyrin-G-specific antibody (Zymed). Note the Flag-tag-specific immunoreactivity within the ankyrin-G positive AIS stretch (arrow, yellow). (**F**) IKKβ-Flag and ankG-eGFP vectors were transfected into mouse cortical neurons as above. 2 days following transfection neurons were cytosol-extracted for visualization of cytoskeletal-associated proteins by incubation in 1% (m/V) Triton-X-100 buffer for 5 minutes. Extractions were performed using pre-cooled solutions and performed at 4 °C, prior to fixation and immunostaining. Note that overfixation often obliterates AIS-immunoreactivity. The inset shows a magnified view of the anti-Flag positive AIS. Note that ankG-eGFP and anti-Flag reactivity remained associated with the non-soluble cytoskeleton in both, in the AIS (arrow), but also in spots within the soma following Triton-X100 extraction. The ankyrin-G antibody showed a notably stronger immunolabeling in the ankG-eGFP transfected cell than in the surrounding untransfected control cells. Scale bars, 10 μm.

To determine subcellular accumulation of NF-κB pathway proteins in the central nervous system, we used gradient centrifugation to separate synaptosomal and lipid fractions derived from the adult mouse cortex. Anti-IKKα/β (inhibitor of kappa light polypeptide gene enhancer in B-cells, kinase) co-localized with anti-NEMO (NF-κB essential modulator) and anti-p65/NF-κB reactivity in ankyrin-G-positive lipid fractions in addition to synaptophysin-positive synaptosomes (Fig. 1A,B). We next determined whether anti-IKKα/β immunoreactivity co-immunoprecipitates with anti-ankyrin-G (AnkG). Following pull-down of ankyrin-G proteins from mouse cortical extracts using anti-ankyrin-G monoclonal and polyclonal antibodies, anti-IKKα/β immunoreactivities were strongly detected, but not found in the lanes of negative control precipitates (Fig. 1C, Suppl. Fig. 1A,C). These findings were validated following reverse immunoprecipitation of IKKβ as well as ankyrinG-eGFP (ankyrinG::enhanced green fluorescent protein) pull-down, which co-immunoprecipitated IKKβ-Flag as detected by anti-Flag antibodies (Fig. 1D–G, Suppl. Fig. 1B). We then investigated how ankyrin-G and IKK-signalosome components co-localized to the cytoskeleton in the axon initial segment following detergent extraction. IKKγ/NEMO and IKKα/β immunoreactivities were retained at the proximal AIS cytoskeleton following Triton X-100- or saponin-mediated extraction of cytosolic proteins (Fig. 2A–D). Following overexpression of Flag-tagged IKKβ we found the protein was also present in the AIS when detected by anti-Flag antibodies. This localization was preserved following detergent extraction, where anti-Flag immunoreactivity co-localized to co-overexpressed ankyrinG-eGFP in the cells’ soma and in the AIS (Fig. 2E,F).

### Interaction of IKKα/β and ankyrin-G *in situ*

**Figure 3:**
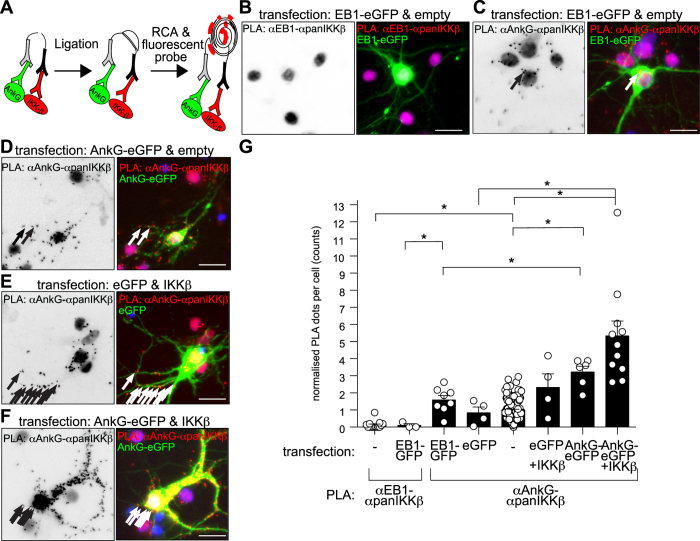
Proximity-ligation assays (PLA) indicate molecular proximity between IKKα/β and ankyrin-G. (**A**) Principle of proximity-ligation assays to detect proximity of the IKK-complex and ankyrin-G. Oligonucleotide-labeled antibodies hybridize and are amplified via rolling-circle amplification when in molecular proximity (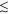
40 nm). (**B**–**E**) Panels depicts mature cultured cortical neurons transfected with eGFP, EB1-eGFP, as negative control or ankG-eGFP with or without IKKβ expression plasmids. PLA between anti-EB1-anti-panIKKα/β as negative control, or anti-ankyrin-G and anti-panIKKα/β epitopes, was conducted using anti-mouse and anti-rabbit specific antibodies. Arrows depict PLA-reactivity in the axon. The experiment was repeated under similar conditions with comparable results. (**G**) Somatic and proximal PLA-spots were counted from 3-183 transfected and un-transfected cells pooled from 1–2 experiments. Kruskal-Wallis, Dunn’s *post*-*hoc* test. Scale bars 10 μm.

To further analyze whether anti-IKKα/β epitopes were found in molecular proximity (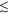
40 nm) to ankyrin-G epitopes, we employed proximity-ligation assays (PLA, Schematic Fig. 3A). Following plasmid transfection, we found significantly more anti-ankyrin-G & anti-IKKα/β PLA-derived spots in ankG-eGFP plus IKKβ overexpressing cortical neurons than we found in control transfected neurons, and notably more than we found in ankG-eGFP or IKKβ plus eGFP expressing neurons or neurons transfected with the unrelated EB1-eGFP vector (Fig. 3B–G), overall suggesting antigen-specific reactions.

### Gene silencing of ankyrin-G increases, while overexpression of ankyrin-G decreases NF-κB dependent reporter-gene activity

**Figure 4:**
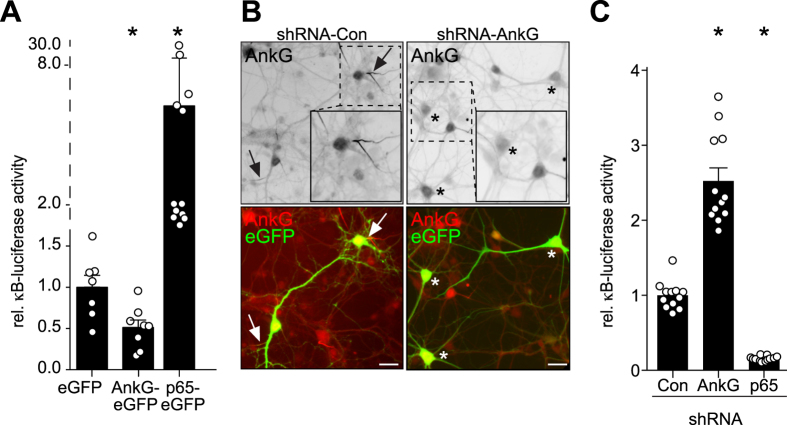
Ankyrin-G negatively regulates NF-κB reporter-gene activity. (**A**) AnkG-eGFP inhibits neuronal constitutive NF-κB^61^ activity. Mature cortical neurons were transfected with ankG-eGFP or p65-eGFP and κB-dependent luciferase reporter activity was monitored in Dual-Luciferase assays (n = 13–19 wells pooled from 3 experiments, Kruskal-Wallis followed by Dunn’s test). (**B**) Efficient suppression of ankyrin-G expression at the axon initial segment following transduction with ankyrin-G-specific small-hairpin RNA. Mature cortical neurons were transfected with ankyrin-G shRNA or control vector together with eGFP for 5 days and immunolabeled. Scale bar, 10 μm. Note transfected ankyrin-G positive AIS marked by arrow or negative cell bodies marked by asterisk. One transfected cell is shown magnified in the boxed area. (**C**) Depletion of ankyrin-G expression in mature cortical neurons induces NF-κB activity. Mature cortical neurons were transfected with ankG- or p65-specific shRNA together with Dual-Luciferase reporter plasmids for 5 days and κB-dependent reporter gene expression was determined (n = 12 wells pooled from 2 experiments, Kruskal-Wallis, Dunn’s *post*-*hoc* test, experiment was repeated 2 times with equivalent results).

**Figure 5:**
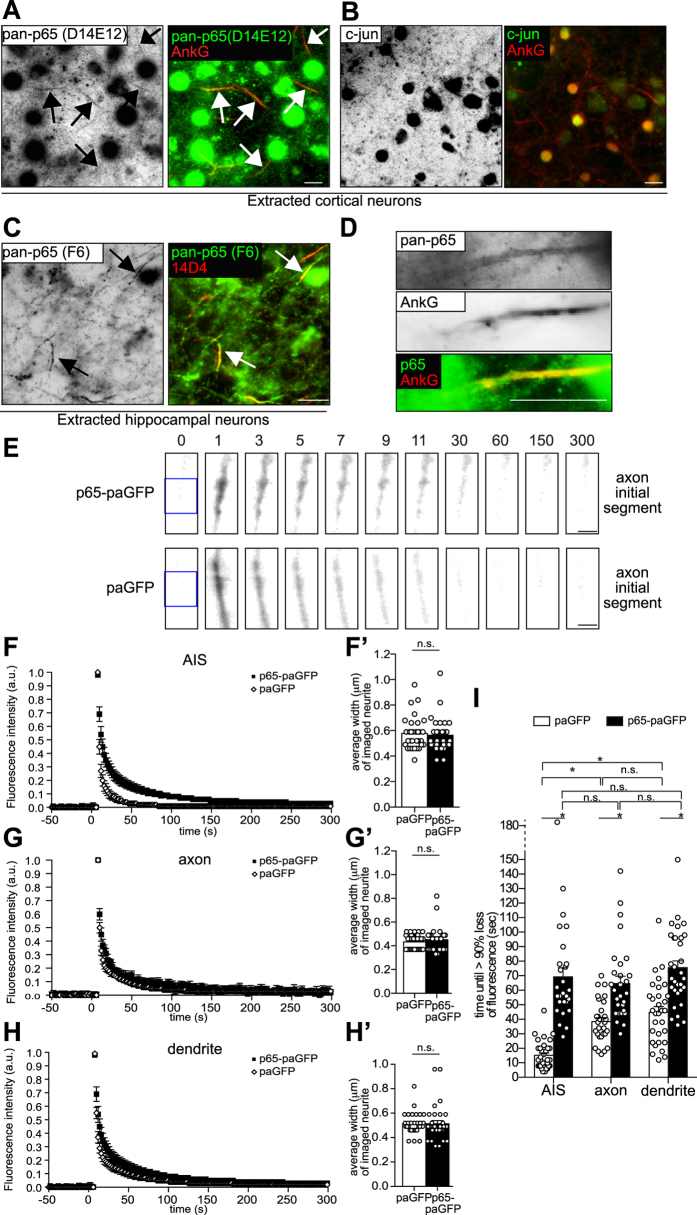
The transcription factor p65/NF-κB localizes to the axon initial segment. (**A**–**D**) Mature cortical neurons were cytosol-extracted using a Triton-X100-based buffer. Extractions were performed at 4 °C. Then, neurons were either immunolabeled using a rabbit monoclonal anti-p65 (green, D14E12, CST), a rabbit monoclonal anti-cJun (green, Cell Signaling Technology) or a mouse monoclonal p65/NF-κB-specific antibody (green, pan-p65, F-6, SCBT) together with either mouse monoclonal (413) or rabbit polyclonal ankyrin-G-specific (H-215, both SCBT) antibodies. AIS highlighted by arrows. (D) Experiment was conducted as in C, with single AIS magnified. Note the labeling of pan-p65 immunoreactivities along the axon initial segment. Color overlay is depicted following background substraction. Scale bars, 10 μm. (**E**) p65-paGFP exhibits decreased mobility in the AIS. Following photo-activation of p65-paGFP or paGFP in DIV12–19 rat hippocampal neurons, fluorescence intensity decline was monitored. Representative time course images of photo-activated p65-paGFP or paGFP in the AIS are depicted. DsRed2 fluorescence (not shown) was used to identify the axon initial segment of transfected neurons based on morphological criteria. Times indicate time points after photo-activation. Regions marked with blue boxes were irradiated with 405 nm light to photo-activate the paGFP/p65-paGFP. Note that 1 second after photo-activation, p65-paGFP fluorescence increase is mainly confined to the irradiated region, while paGFP fluorescence has already spread over the region’s boundaries. Scale bars, 2 μm. (**F**–**H**) Traces of mean fluorescence intensity of p65-paGFP and paGFP in the irradiated region ±SEM, n = 3–4 experiments, per experiment 6–12 neurons were analyzed. (**I**) Bar graphs depict average time point of over 90% fluorescence intensity reduction after photo-activation in 28–33 neurons per group in 3–4 different platings. Time points were determined from data represented in the traces. Neurites of similar morphology and widths were imaged (**F**’–**H**’). No significant differences were found between paGFP and p65-paGFP expressing neurons. n.s.: not significant, one-way ANOVA.

IKKα/β is a positive regulator of p65-phosphorylation and NF-κB-dependent transcription. Surprisingly, we found that ankyrin-G overexpression depressed NF-κB activity in mature cortical neurons (Fig. 4A), while ankyrin-G small-hairpin vector-mediated depletion significantly increased NF-κB reporter gene activity over a period of five days (Fig. 4B,C). We also tested the validity of the reporter construct for p65/NF-κB-dependent transactivation by overexpression or knockdown of the transcription factor (Fig. 4A,C). Notably, comparable to IKK-complex immunreactivity (Fig. 2), anti-p65 immunoreactivity was preserved following detergent extraction using two different antibodies (Fig. 5A–D). We additionally determined that an overexpressed p65 photoactivatable-GFP (p65-paGFP) fusion protein exhibits significantly decreased mobility in the AIS when compared to paGFP control. Dynein-dependent microtubule-based transport of p65-eGFP in dendrites was previously reported^27^,^28^. Despite the cytoplasmic diffusion barrier in the AIS^11^,^29^, p65-paGFP mobility in the AIS was comparable to the distal axon and dendrites, suggesting similar retention and active transport mechanisms in all three compartments. paGFP mobility was significantly lower in the neurites compared to the AIS (Fig. 5E–I). Overall, these results suggested that a pool of neuronal p65/NF-κB protein is sequestered by the detergent-insoluble cytoskeleton at the AIS.

### p65/NF-κB binds the ankyrin-3 gene promoter sequence and regulates neuronal ankyrin-G expression via a negative feedback

**Figure 6:**
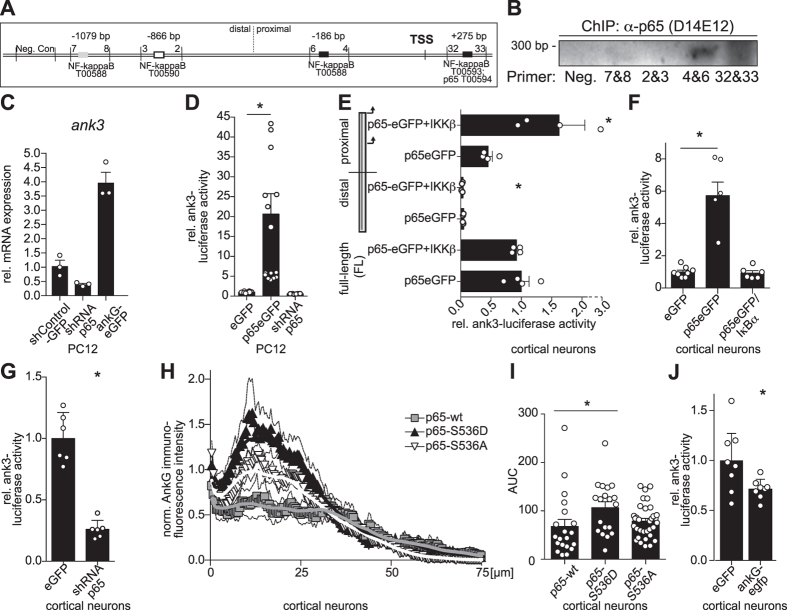
p65 increases *ank3* promoter-dependent reporter-gene activity. (**A**) Schematic drawing of a 2 kB stretch upstream of the putative transcription start site (TSS) of the mouse *ank3* gene containing multiple putative NF-κB transcription factor binding sequences. (**B**) p65/NF-κB binds to the *ank3* gene promoter. p65 ChIP following 2 h TNF-α (100 ng/ml) in mouse BV2 cells, primer numbers surrounding putative NF-κB sequences labeled as in (A). (**C**) qPCR of *ank3* transcripts in PC12 cells following depletion of endogenous p65 protein. PC12 cells were transfected using *p65*-shRNA or ank3-eGFP vectors and analyzed. Fold-induction of *ank3* transcripts was determined using *18S* rRNA-specific primers for normalization (n = 3). (**D**) The *ank3* promoter sequence highlighted in (A) was cloned into a pGL3-luciferase reporter vector and used in Dual-Luciferase-assays following transfection of PC12 cells (n = 8–16, Kruskal-Wallis test, Dunn’s *post*-*hoc*, p = 0.0094). (**E**) TSS proximal promoters bind p65. Proximal and distal parts of above sequence were similarly generated and transfected into mature cortical neurons with IKKβ and p65 expression vectors (n = 4, Mann-Whitney test within fragments, p ≤ 0.05 (proximal and distal)). (**F**) Similarly, p65 induces the *ank3* promoter in primary cortical neurons. These were transfected with p65 and IκBα vectors together with the ankyrin-3-reporter (n = 6–8 wells, Kruskal-Wallis followed by Dunn’s, p = 0.0003). (**G**) Depletion of endogenous p65 suppresses *ankyrin*-*3*-reporter activity. Cortical neurons were transfected using control-/*p65*-shRNA constructs together with the *ank3*-reporter construct for 5 days, relative luciferase activities were determined (n = 6 wells, Mann-Whitney test, p = 0.0022). (**H**) Overexpression of serine536-phospho-mimetic p65-mutants (S536D) results in higher ankyrin-G expression at the AIS than wild-type p65. Ankyrin-G immunofluorescence along the AIS was determined following three days of transfection. (**I**) Quantification of the AUC determined from the single traces obtained from the p65 mutants in ((H) n = 19–33 axons, Kruskal-Wallis test, Dunn’s *post*-*hoc* *p = 0.0093). (**J**) Mature cortical neurons were transfected using the ank3-promoter in conjunction with ankG-eGFP and lysed after two days. Relative ank3-luciferase activity was determined (n = 8 wells, parametric data analyzed by two-sided *t*-*test*, p = 0.00315).

Next, we decided to test whether NF-κB transcriptional feedback controls ankyrin-G expression levels. We focused on a stretch of about 2000 base pairs around the transcription start site (TSS) upstream of exon 1e, expressed in many tissues including the frontal cortex, hippocampus and caudate putamen^5^, of the mouse ankyrin-G (*ank3*) gene. The region was identified on ENSEMBL (ENSMUSR00000028730) and the eukaryotic promoter database (‘*Ank3*_*1*’, epd.vital-it.ch, ref. 30). We examined the sequence for potential p65/NF-κB binding sequences using an established promoter search algorithm (<15% dissimilarity margin, TESS, Fig. 6A). Contained in this area we found 4 potential binding sites for p65/NF-κB. Following brief stimulation of a mouse cell line with tumor necrosis factor-α for appropriate NF-κB induction, we used primer pairs surrounding the previously identified areas in chromatin immunoprecipitation assays (ChIP) using a rabbit monoclonal antibody raised against p65/NF-κB tested for application in ChIP (D14E12). We obtained pronounced amplification from two regions containing potential NF-κB binding sites proximal to the TSS (‘4&6’, ‘32&33’, Fig. 6B). NF-κB/p65 dependent regulation of *ank3* transcripts was determined by depletion of p65 in PC12 cells. Abundance of transcripts was decreased by small-hairpin mediated reduction of p65 protein levels, while ankG-eGFP overexpression confirmed primer specificity (Fig. 6C). We then inserted the *ank3* promoter sequence upstream of a firefly-luciferase reporter gene in order to test whether p65/NF-κB induced *ank3*-promoter dependent reporter activity. p65-cotransfected PC12 cells showed a pronounced and significant up-regulation of luciferase activity from the above reporter construct which was not observed following eGFP transfection or p65 reduction (Fig. 6D). Upon further split into proximal and distal promoter constructs (Schematic Fig. 6A), we found pronounced p65-dependent and IKKβ-potentiated activity from the proximal region mainly in primary cortical neurons, in agreement with the results from the ChIP analyses (Fig. 6E). Additional experiments using the full-length promoter construct in primary cortical neurons confirmed the previous results obtained from PC12 cells (Fig. 6F), and showed that basal ank3-reporter gene activity was diminished in p65-depleted cells (Fig. 6G). We also tested whether transfection of phospho-site mimetics potentiates ankyrin-G protein expression at the AIS of primary neurons. Interestingly, following a 3-day period of expression, we found a significant increase of the ankyrin-G intensities by transfection of p65-S536D mutants (Fig. 6H,I). Consistent with decreased p65/NF-κB activity following overexpression of ankyrin-G, we found notably decreased ank3-dependent reporter activity following overexpression of the scaffolding protein (Fig. 6J).

## Discussion

Neuronal plasticity allows the nervous system to adapt to intrinsic and extrinsic cues. Recent evidence suggests a novel form of plasticity owing to alterations of positioning and length of the axon initial segment (AIS)^31^,^32^. The AIS integrates information from somatodendritic, axo-axonic and inhibitory input, and computes axonal output based on the molecular make-up and positioning of this compartment^8^,^31^,^33^. These properties may explain its recently emerging involvement in the pathophysiology of neurodevelopmental disorders such as Angelman syndrome^34^, bipolar disorder^35^,^36^,^37^, intellectual disability^38^ and posttraumatic stress disorder^39^ (reviewed in ref. 40).

The transcription factor NF-κB was found in the axoplasm^41^,^42^,^43^. Previous studies described an accumulation of phosphorylated NF-κB pathway proteins at the AIS of rodents and humans *in vitro* and *in vivo*^44^. Immunoreactivity to phosphorylated IκBα and activated IKK has been found at nodes of Ranvier^45^ and both proteins were implicated in axon outgrowth^46^. Despite the proposed key role of the AIS in neuronal physiology and pathophysiolgy, little is known about the transcriptional regulation of its main components. Here, we found that ankyrin-G and IKKα/β co-localized in membrane and synaptosomal fractions of mouse cortical extracts and co-immunoprecipitated from cortical lysates, also evidenced in the reverse co-immunoprecipitation and pull-down experiments. Membranes were extracted using the mild detergent IGEPAL CA-630, as previously employed in co-immunoprecipitation work on the membrane-associated ankyrin-spectrin network^47^. In IκBα knockout mice persistent staining for phosphorylated IκBα^48^ was shown, raising concerns regarding phosphorylation-specific antibody specificity. A potential compensatory up-regulation or cross-reactivity to phosphorylated IκB isoforms have not been determined in the cited study. Whole protein, pan-specific polyclonal antibodies are considered less promiscuous compared to phosphorylation-specific antibodies. Here we found that the IκBα-upstream kinase complex associated with detergent-insoluble cytoskeletal compartments and demonstrate that pan-IKKγ and pan-IKKα/β co-localized with ankyrin-G. Previous studies suggested that IKKγ/NEMO also localized to the AIS cytoskeleton following detergent extraction, and demonstrated that it interacted with AIS-localized FHF1/FGF12 (fibroblast growth factor homologous factor 1)^26^. Furthermore, IKK-complex inhibitors also inhibited the interaction of sodium channels with FHF4/FGF14, both also localized at the AIS^49^,^50^. Collectively, these data suggest that IKK signaling components localize to the AIS.

Importantly, the co-localization of ankyrin-G and IKKα/β was consolidated in proximity-ligation assays, thus validating their proximity in the molecular range (smaller than about 40 nm^51^). This interaction significantly increased following overexpression of both proteins. Ankyrin-G contains ankyrin- and death-domains (DDs) that may serve as scaffolds upstream of NF-κB activation. DD interactions form platforms that either result in apoptosis^52^ or signal towards NF-κB. Depending on the nature of the scaffold they either decrease^53^ or potentiate transcription factor activity^54^. Interestingly, we here show that ankyrin-G-eGFP expression decreased NF-κB activity in cortical neurons, suggesting that p65 availability is reduced following sequestration by the ankyrin-repeats of ankyrin-G in a manner similar to IκBα-ankyrin-repeat domain sequestration of the p65-Rel-homology domain^55^. In turn we found that increased levels of p65 activated the NF-κB-reporter following silencing of ankyrin-G. We also found p65 to be associated with the AIS cytoskeleton. p65 immunoreactivity was previously described in the axoplasm^41^,^42^,^43^ and was activated following sciatic nerve transection^56^. In further studies, we found that *ank3* transcripts and reporter-gene expression were regulated in an NF-κB dependent manner, thus potentially allowing for a modulation of ankyrin-G length and position along the AIS^9^,^31^,^32^,^57^ and its expression levels at spine heads^58^. Database queries suggest several transcription start sites for the rodent *ank3* gene. Ankyrin-G isoform expression may be regulated not only through alternative splicing but also through alternative start sites. Notably, shorter variants occur post-synaptically and help in the organization of nanodomains at glutamatergic synapses^58^. It has previously been demonstrated that ischemic injury resulted in the disassembly of the ankyrin-G-related AIS-cytoskeleton, which began at early time-points following the insult, and was inhibited by a calpain inhibitor^17^. Interestingly, neurons that were completely devoid of the AIS diffusion barrier failed to re-establish a functional AIS. More recent work implicated P2X7 receptors in the calpain-dependent ankyrin-G depletion following CNS insults^59^. Our findings suggest that increasing the activity of the transcription factor NF-κB may up-regulate a set of ankyrin-G transcripts and protein under such pathophysiological conditions, thereby preventing injury-induced AIS disassembly and promoting spine maintenance.

In conclusion, we demonstrate that p65/NF-κB controls ankyrin-G levels via a negative feedback loop, thereby linking NF-κB signaling with neuronal polarity and axonal plasticity. Constitutive NF-κB activity may help to maintain ankyrin-G expression levels in differentiated neurons, while sequestration of the transcription factor by ankyrin-G may restrict NF-κB overactivity and overabundance of ankyrin-G. This feedback loop may also be appropriately positioned to maintain expression levels of ankyrin–G during pathophysiological conditions.

## Methods

### Chemicals

Chemicals came from Sigma-Aldrich (Wicklow, Ireland) or Merck Chemicals (Nottingham, UK), unless stated otherwise. Primers for PCR and cloning applications were obtained from Sigma-Aldrich (Wicklow, Ireland). Restriction enzymes were from Fermentas (Life Technologies, Dun Laoghaire, Ireland) or New England Biolabs (New England Biolabs, Brennan&Co., Stillorgan, Ireland). TNF-α was from PeproTech, London, UK. Cell culture media was purchased from Gibco-Invitrogen (Dun Laoghaire, Ireland) and pre-designed p65-HuSH-29 small-hairpin RNA-vector constructs from Origene (Rockville, MD, USA).

### Cell Culture

Phaeochromocytoma (PC)12 cells were cultured in RPMI media including 10% Horse serum, 5% Fetal Bovine Serum (FBS), 2 mM L-Glutamine and 1% Penicillin Streptomycin; SH-SY5Y cells were cultured in DMEM/F-12 media, including 10% FBS, 2 mM L-Glutamine and 1% Penicillin/Streptomycin. Cells were kept at 37 °C in 5% CO_2_ in a humidified atmosphere and passaged every 3–5 days. PC12 and SH-SY5Y cells were transfected using Lipofectamine 2000 (Gibco-Invitrogen) or Transit-X2 (Mirus, Madison, WI, USA) or nucleofection (Lonza, Tewkesbury, UK) and using standard protocols. Primary cultures from *C57BL*/*6* E16 mouse pups were obtained from pregnant mothers, the cortices were isolated, minced and following trituration and trypsin-digest for 30 mins at 37 °C, they were cultured for one day in DMEM, 10% FBS, 2 mM Glutamine and then in Neurobasal (Gibco), 2% B27, 1% GlutaMAX (Gibco), or NMEM-B27 media (1xMEM, 1 mM sodium pyruvate, 26 mM NaHCO_3_, 2 mM GlutaMAX, 1xB27 and 33 mM β-D-Glucose), including β-D-arabino-furanoside (0.6 μM, DIV1–2), for the days-*in*-*vitro* (DIV) as indicated. Transfection of primary mature neurons was performed using calcium precipitation as previously described^26^,^60^. Transfections were conducted on mature cortical neurons using pGFP-EB1 (Addgene Plasmid #17234); pFlag-IKK-2 (IKKβ; Addgene #11103); pAnkG-eGFP^5^ (270 kDa; a kind gift of V. Bennett, Duke University Medical Center, Durham, NC, USA); p65-eGFP (a generous gift of E. Floettmann and M. Rowe, Cardiff, UK); IκBα-wt (a kind gift of F. Peyron, Inserm 364, Nice, France); p65-wt, p65-S536D, p65-S536A (a generous gift of Carl Y. Sasaki, NIH, Baltimore, MD, USA).

Animal procedures were carried out under a license from the Department of Health and Children of Ireland (B100/3688) and the Health Products Regulatory authority (HPRA, AE19127/P005), and were previously approved by the Research Ethics Committee of the Royal College of Surgeons in Ireland (REC131 & 817).

### Immunofluorescence and live-cell analyses

For fixation glass-sealed formaldehyde solutions (Mallinkrodt, Dublin, Ireland) were freshly diluted from 16% to 3% content using 1X cytoskeletal buffer (CB buffer: 10 mM PIPES (pH 6.8), 150 mM NaCl, 5 mM EGTA, 5 mM glucose, 5 mM MgCl_2_). Following recovery of cell culture media, freshly prepared and pre-warmed (37 °C) formaldehyde solutions were added directly to cells, wells were sealed and incubated at 37 °C for 12–20 minutes. Following washes in cold HBSS, cells were permeabilized using ice-cold HBSS containing 0.1% (w/v) Triton X-100, blocked with 0.3% (w/v) Triton X-100 and 5% (v/v) horse serum in HBSS. They were incubated for 2 hours at RT or overnight at 4 °C in primary antibody dissolved in 0.3% (w/v) Triton X-100, 1% (v/v) horse serum in HBSS. The following primary antibodies were used, mouse monoclonal anti-ankyrin-G (H-4 & 463, 1:500, Santa Cruz Biotechnology (SCBT), Heidelberg, Germany, Cat# sc-12719, RRID:AB_2057712; Cat# sc-12719, RRID:AB_626674), a rabbit polyclonal anti-ankyrin-G (H-215, 1:1000, SCBT Cat# sc-28561, RRID:AB_633909), a mouse monoclonal ankyrin-G-specific antibody (Thermo Fisher, Paisley, UK, Cat# 33-8800, RRID:AB_2533145, 1:500), a rabbit polyclonal anti-Flag-specific antibody (Sigma, Cat# F7425, RRID:AB_439687, 1:500), an anti-(pan)-IKKα/β, (H-470, 1:1000, SCBT, Cat# sc-7607, RRID:AB_675667), anti-(pan)-IKKγ/NEMO (1:200, Abcam,Cambridge, UK, Cat# ab77750, RRID:AB_1566331), a rabbit monoclonal anti-p65 (D14E12, Cell Signaling Technology (CST), Leiden, Netherlands, Cat# 8242, RRID:AB_10859369, 1:500), a mouse monoclonal p65/NF-κB-specific antibody (pan-p65, F-6, SCBT, Cat# sc-8008, RRID:AB_628017, 1:500), a rabbit monoclonal anti-c-Jun (60A8, CST, Cat# 9165, RRID:AB_2130165, 1:500) as well as the antibody 14D4 (rabbit polyclonal, #2859, CST, Cat# 2859, RRID:AB_561111, 1:500). Following three washes in ice-cold HBSS, cultures were incubated for 1 hour at RT with secondary antibodies raised against the appropriate species and tagged with either Alexa–Fluor-488 or -568 (Gibco-Invitrogen). Photomicrographs were obtained using a SPOT RT SE 6 Camera (Diagnostic Instruments, Sterling Heights, USA) on an Eclipse TE 300 inverted microscope (Nikon, Kinston upon Thames, UK) and on a Te2000-s with 40x magnification with Mercury-arc light bulbs. A Zeiss 510 or 710 LSM (Zeiss, Jena, Germany) was used for confocal image analyses. Images were processed and analyzed using ImageJ (1.50 f, NIH, Baltimore, USA) and MetaMorph software (version 7.0 and higher, Molecular Devices, Berkshire, UK). Background subtractions were applied using the same settings to all images of an experimental image set, where indicated and using the ImageJ software routine rolling ball or the Metamorph flatten background routine. Cytosolic extractions were performed by incubation of cells for 5 minutes in 1% (m/V) Triton-X-100 or 0.5% saponin in 10 mM Na_3_PO_4_-buffer (pH 7.4), 1 mM MgCl_2_, 3 mM CaCl_2_, 150 mM NaCl. Pre-cooled solutions were used and the procedure was performed at 4 °C, prior to washes in PBS, fixation and immunostaining. Photoactivation experiments were conducted using photoactivatable eGFP (a kind gift of G.Patterson and J. Lippincott-Schwartz, The National Institute of Child Health and Human Development, NIH, MD USA) on a LSM 5 live Duo confocal microscope. The p65 sequence was cloned from a human p65-eGFP vector into a vector encoding photoactivatable eGFP using *Hind*III and *Bam*HI. Neurons were transfected via calcium phosphate precipitation with vectors encoding p65-paGFP or paGFP, as well as the red fluorescent protein DsRed2. During photo-activation measurements, neurons were maintained in in a buffer (120 mM NaCl, 20 mM HEPES, 12 mM glucose, 5 mM NaHCO_3_, 3.5 mM KCl, 1.2 mM CaCl_2_, and 0.4 mM KH_2_PO_4_; pH 7.4) supplemented with 1.2 mM MgCl_2_, at 37 °C and atmospheric CO_2_. paGFP was excited using a 489 nm laser diode (100 mW, 4% laser power) and light was collected through a 495–555 nm bandpass filter. DsRed2 was excited at 561 nm (40 mW, 4% laser power) and emissions were collected through a 575 nm long pass filter. For paGFP activation, a 3.3 μm long region was illuminated with 405 nm light from a laser diode (50 mW, 100% laser power) for 20 iterations, in total 0.78 s. Images of paGFP fluorescence were acquired every 2 s and measurements started 1 min before photo-activation, while finishing 5 min after photo-activation. paGFP fluorescence intensity was background subtracted and the resulting traces were corrected for baseline slope. For comparison of traces, maximum paGFP fluorescence intensity values were normalized to one, while baseline fluorescence was set as zero. Resulting curves of fluorescence loss after photo-activation were fitted using equations of second order exponential decay.

### Proximity-ligation assays

Proximity-ligation assays to detect antigens in molecular proximity were conducted on carefully formaldehyde-fixed primary cortical neurons. Briefly, following standard immunofluorescence procedures including primary antibody incubation, a rabbit anti-IKKα/β (SCBT, Cat# sc-7607, RRID:AB_675667, 1:500), anti-EB1 (BD Biosciences, Oxford, UK, Cat# 610534, RRID:AB_3978911:500) or a mouse monoclonal anti-ankyrin-G (SCBT, Cat# sc-12719, RRID:AB_626674, 1:500) were used overnight, incubation of Olink-specific plus/minus oligonucleotide-linked antibodies in antibody-dilution buffer followed (Duolink, Olink Bioscience, Uppsala, Sweden). Hybridization of oligonucleotides and detection was conducted as advised by the manufacturer’s instructions.

### Gradient centrifugation for subcellular fractionation

Brains of adult *C57BL*/*6* mice were dounced and minced in 5 ml STE-buffer (0.32 M Sucrose, 10 mM Tris, 1 mM EDTA, pH 7.2–7.4, including protease and phosphatase inhibitor cocktails (1:100, Sigma)) per brain. 10% and 7.5% Ficoll solutions (Sigma) were prepared in ice-cold STE-buffer from 20% Ficoll stock solution and carefully layered. The brain lysates were centrifuged at 1200 *g* for 5 minutes, the pellet labeled nuclei was immediately lysed in 5 M urea-SDS-lysis buffer. The supernatant of the above step was spun at 11,100 *g*, the pellet re-suspended and layered on top the Ficoll gradient. Gradient centrifugation followed at 100,000 *g* for 30 minutes using ultracentrifugation (Sorvall, Thermoscientific, UK) and a lipid fraction, a synaptosomal fraction as well as a mitochondrial and nuclear fractions were isolated and lysed in SDS-lysis or 5 M-urea buffer for Western-blot procedures.

### Immunoprecipitation and Western-blotting

For immunoprecipitation, brains were minced using dounce-homogenization in ice-cold sodium-sucrose buffer, 140 mM NaCl, 2 mM EDTA, 20 mM Tris (pH 7.4), 250 mM sucrose, 10 mM β-glycero-phosphate, 50 mM NaF, including protease inhibitor cocktail and phosphatase inhibitor cocktail 2&3 and 1% IGEPAL CA-630. Cells were briefly rinsed with ice-cold HBSS (Gibco) including protease and phosphatase inhibitors (Sigma-Aldrich; 1:1000), prior to lysis for 1 h on rotation. Lysates were cleared by centrifugation at 13,000 rpm for 10–20 minutes before protein determination and immunoprecipitation. We used 250–500 μg of protein lysate as indicated with 5–25 μl of rabbit polyclonal anti-ankyrin-G (a kind gift of S.E. Lux, Children’s Hospital, Boston, USA), rabbit polyclonal or mouse monoclonal anti-ankyrin-G (H-215; clone463, SCBT) antibody, rabbit polyclonal anti-MAP2 (sc-20172, SCBT, microtubule-associated protein-2), or anti-IKKα/β (H-470) antibodies in Na-Sucrose lysis buffer following pre-clearing of the lysates and linkage to protein A/G-Agarose beads (SCBT). For GFP-pulldown, following transfection of SH-SY5Y cells and lysis in Co-IP buffer (50 mM Tris·Cl, pH 7.5, 15 mM EGTA, 100 mM NaCl, 0.1% (w/v) Triton X-100), 5 or 1 μg of a mouse monoclonal anti-GFP (B-2, SCBT, Cat# sc-9996, RRID:AB_627695) was used with 100 μl protein A/G-agarose beads or 50 μl dynabeads protein-G. IKKβ-Flag was detected using a rabbit polyclonal anti-Flag (OctA, SCBT, Cat# sc-807, RRID:AB_675756, 1:2000), ankG-eGFP-fusion protein by a rabbit monoclonal anti-GFP antibody (E385, Abcam, Cat# ab32146, RRID:AB_732717, 1:2000). Immunoprecipitates were washed in lysis buffer and PBS and solubilized in SDS-loading buffer for subsequent Western-blot analyses using standard techniques. For Western-blot analyses cells were lysed in RIPA buffer (150 mM NaCl, 1.0% IGEPAL CA-630, 0.5% Na-deoxycholate, 0.1% sodium dodecyl sulfate, 50 mM Tris-HCl, pH 8.0, including protease and phosphatase inhibitor cocktail). Following standard procedures for cell lysis and protein determination, equal amounts of protein were separated by 6–15% gel electrophoresis as appropriate and immunoblotted using semi-dry transfer. Following blocking procedures, antibodies were dissolved in Tris-buffered saline, 0.05% Tween-20 and 3–5% semi-dry milk. The following antibodies were used in Western blotting, rabbit anti-ankyrin-G (Lux), mouse monoclonal anti-ankyrin-G (463, SCBT) antibody, anti-NEMO (1:1000, #ab77750, Abcam), rabbit polyclonal anti-ankyrin-G (H-215), anti-pan-p65 (1:5000, F-6X, SCBT, 1:500), rabbit anti-IKKα/β (1:1000, H-470, SCBT), mouse monoclonal anti-α-Tubulin (1:5000, Sigma), mouse monoclonal anti-GAPDH (1:1000, 6C5, Abcam, Cat# ab8245, RRID:AB_2107448), anti-synaptophysin (Sigma, Cat# SAB4502906, RRID:AB_10746692), rabbit polyclonal anti-Lamin A/C (CST, Cat# 2032, RRID:AB_2136278) and a rabbit polyclonal anti-VDAC2 (1:1000, Abcam. Cat# ab37985, RRID:AB_778790). Detection was conducted using appropriate HRP-linked secondary antibody detection (Jackson Immunoresearch, Newmarket, UK).

### Molecular cloning of mouse *ank3*-*promoter* firefly luciferase reporter gene and small-hairpin constructs directed at mouse *ank3* mRNA and qPCR procedure.

The following primers were used to PCR amplify mouse *ank3* gene specific reporter-gene constructs from mouse total DNA preparations. 5′-GCTAGCTAGCCTTTCCTTGAGATCAGGGGAAGT-3′ and 5′-TTAGGTACCGGGTGGGGCAACCGC-3′, with a PCR product size of 1903 bp, which were cloned into pGL3-Basic vector (Promega) using the restriction enzymes, *KpnI* and *NheI* (Fermentas, Life Technologies, Dun Laoghaire, Ireland). The amplified sequence is located directly upstream and contained in the region encoding exon 1e (ref. 5), expressed in ankyrin-G transcripts of approximately 93 kDa, 192 kDa, 209–214 kDa and also 500 kDa (Addgene Plasmid #86436). Primers for obtaining the first and second half segments of the promoter were used in conjunction with the above primers to obtain these promoter constructs as follows, 5′-GCTAGCTAGCCTGGCTTTTTCTGTGCCAGAA-3′ and 5′-TTAGGTACCTTCTGGCACAGAAAAAGCCAG-3′. Amplified sequences were verified by sequencing using RV3 standard sequencing primers (MWG, Germany). For preparation of the *ankyrin*-*3* mRNA directed constructs we used the previously published^2^ sequence ‘CT19’ -5′-GAGACATAAACTGGCCAAC-3′ cloned into pSilencer2.1. qPCR for *ank3* mRNA was conducted following Qiagen RNeasy plus - aided (Qiagen, Hilden, Germany) RNA isolation, reverse transcription using random hexamer primers and using the following primers, rat *ank3*, FW 5′-GTGCATTCTGGGTTTCTGGT-3′, REV 5′-CACTATGACCGGGCCTAAAA-3′ *18S*, FW-5′-GTAACCCGTTGAACCCCATT-3′, REV 5′-CCATCCAATCGGTAGTAGCG-3′ on a Roche Lightcycler 2.0 (Roche, Penzberg, Germany) with SYBRgreen (Quantitect, SYBRgreen kit, Qiagen) using manufacturers’s instructions.

### Chromatin-Immunoprecipitation (ChIP) analysis

Cell culture of a murine BV-2 cell line was conducted using RPMI, 10% FBS, 2 mM Glutamine, 1% Penicllin/Streptomycin, the cells were stimulated using 100 ng/ml rmTNF-α (PeproTech, London, UK), 2 hours before fixation in 1% formaldehyde, left at 37 °C for 10 min and quenched with 2 mM Glycine. Following pellet washes, resuspension in sonication buffer followed (1% Triton X-100, 0.1% Na-deoxycholate, 50 mM Tris-Cl, pH 8.10, 150 mM NaCl, 5 mM EDTA, 0.1% PMSF, 1% SDS and protease inhibitor cocktail) and sonication on full power for 3 × 10 sec on ice (Branson Digital Sonifier 250, Danbury, USA). The cells were spun at 13,000 rpm for 2 min. Supernatants underwent agarose gel electrophoresis for fragment length check (400–600 bp) and were used for immunoprecipitation. To protein A/G-agarose, FBS and Fish Sperm DNA (Sigma) was added and incubated for 30 mins for preclearing, with 5 μl of rabbit monoclonal anti-p65 (D14E12, CST) for immunoprecipitation overnight at 4 °C. Samples were spun and underwent series of washing steps before extraction of DNA using 1% SDS, 0.1% NaHCO_3_. The samples were incubated at 65 °C for 2–4 hours and purified using a PCR purification kit. PCR was conducted using standard procedures at 45 cycles using the following primer pairs (5′ -> 3′): ‘7’-CTGGCCTTCTGGGTCTCATTTACGC & ‘8’-CGGGTGTGGTCGCTTAAGCATCTTT, ‘3’-TTAAGCGACCACACCCGACAGT & ‘2’-CAAAAGGTTCAGCCCCTGGCTTT, ‘4’-CGTTACCACCCCCACTGCTG & ‘6’-AAAGGGGGAGGCATACGTGCAT, ‘32’-GGAAGGAAGAAAACAAAGGGTTGGAA & ‘33’-CAGGGGAGTTGAGACGAGGC.

### Statistical analysis

We employed GraphPad Prism for statistical analyses (GraphPad Software Inc., La Jolla, USA). Significance was determined following parametric testing and using the test as detailed in the figure legends. All data are represented as mean ± SEM, data values are represented by a circle, p values ≤ 0.05 were considered to be significantly different and marked by an asterisk.

## Additional Information

**How to cite this article**: König, H.-G. *et al*. NF-κB regulates neuronal ankyrin-G via a negative feedback loop. *Sci. Rep.* **7**, 42006; doi: 10.1038/srep42006 (2017).

**Publisher's note:** Springer Nature remains neutral with regard to jurisdictional claims in published maps and institutional affiliations.

## Supplementary Material

Supplementary Figure 1
