# Supplementary material for: NF-κB regulates neuronal ankyrin-G via a negative feedback loop
Source: Sci Rep. 2017 Feb 9;7:42006. doi: 10.1038/srep42006 (PMC5299403; doi:10.1038/srep42006)
Supplement: Supplementary Figure 1 [file srep42006-s1.pdf]

Scientific Reports 2016

Supplementary Figure 1

"NF-κB regulates neuronal ankyrin-G via a negative feedback loop"

Hans-Georg König, Robert Schwamborn, Silke Andresen, Sinéad Kinsella, Orla Watters, Beau Fenner & Jochen H. M. Prehn

Supplementary Figure 1:

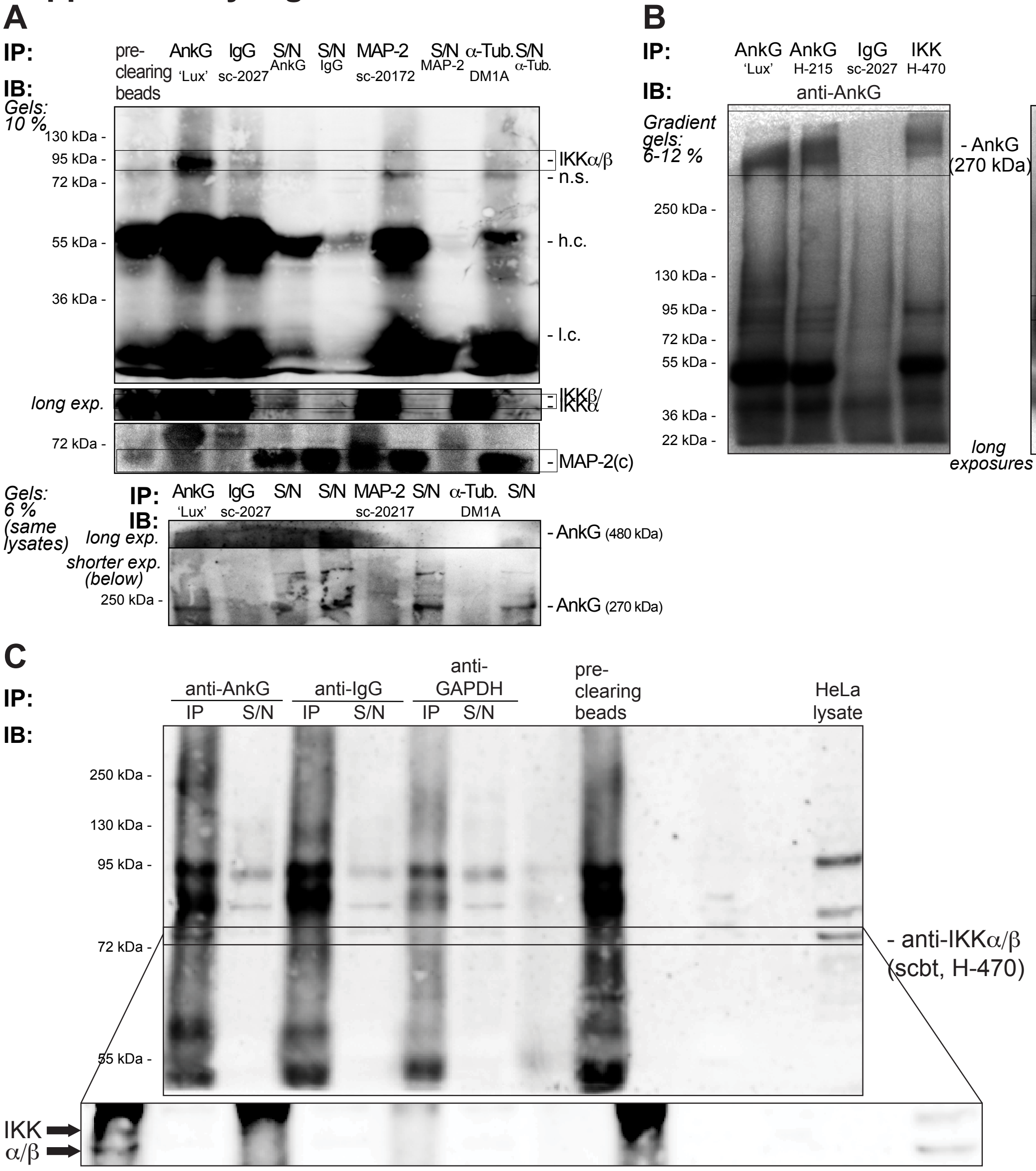

Supplementary Figure 1. Ankyrin-G immunoprecipitation experiment overviews. (A-C) For immunoprecipitation, mouse brains were minced using dounce-homogenization in ice-cold sodium-sucrose buffer (140 mM NaCl, 2 mM EDTA, 20 mM Tris (pH 7.4), 250 mM sucrose, 10 mM β-glycero-phosphate, 50 mM NaF, including protease inhibitor cocktail and phosphatase inhibitor cocktail 2&3 and 1% IGEPAL CA-630). Cells were briefly rinsed with ice-cold HBSS (Gibco) including protease and phosphatase inhibitors (Sigma-Aldrich; 1:1000), prior to lysis for 1 h on rotation. We used 250-500 μg of protein lysates. Immunoprecipitates were washed and solubilized in SDS-loading buffer lysis buffer for subsequent Western-blot analyses using standard techniques. For Western-blot analyses lysis was in RIPA buffer (150 mM NaCl, 1.0% IGEPAL CA-630, 0.5% Na-deoxycholate, 0.1% sodium dodecyl sulphate, 50 mM Tris-HCl, pH 8.0, including protease and phosphatase inhibitor cocktail). **(A,B)** Whole blot views and alternative exposure times of the experiments depicted in Fig. 1B&C. Here, we also included all additional controls performed (e.g. S/N, supernatants of immunoprecipitations), and provide information on antibody clones/catalogue numbers used and percentages of electrophoresis gels employed. The following antibodies were used for immunoprecipitation: rabbit polyclonal anti-ankyrinG (a kind gift of S.E. Lux), rabbit polyclonal anti-ankryinG (SantaCruz), rabbit IgG (SantaCruz); rabbit polyclonal anti-MAP-2 (SantaCruz), mouse monoclonal anti α-Tubulin (SantaCruz), rabbit polyclonal anti-IKKα/β (SantaCruz). Note the prominent recactivity for IKK-α/β (H-470, scbt) at the appropriate molecular weight between the 72 and 95 kDa markers, note also the IKK-reactivity following longer exposures in the supernatants between the same markers. Also note the immunoreactivities against ankG (detected by clone 463, scbt) and at the top of the gel (following longer exposure, representing the 480 kDa isoform, detected by clone 463, scbt). Abbreviations used: h.c./l.c., heavy/light chain, n.s., non-specific band. **(C)** Additional immunoprecipitation experiment performed following immunoprecipitation from rat neuronal extracts using mouse monoclonal anti-ankyrin-G (463; sc-12719), or anti-IgG and anti-GAPDH (6C5, Abcam) antibodies. Depicted is the detection using anti–IKKα/β-specific antibodies (SantaCruz), find the latter magnified in a panel below for better visualization. As positive control for IKK-reactivity human HeLa cell lysates (cultured using standard techniques) were included and depicted in the last lane.
